# Influence of Transcranial Direct Current Stimulation and Exercise on Fatigue and Quality of Life in Multiple Sclerosis

**DOI:** 10.3390/healthcare11010084

**Published:** 2022-12-28

**Authors:** Inés Muñoz-Paredes, Azael J. Herrero, Natalia Román-Nieto, Alba M. Peña-Gomez, Jesús Seco-Calvo

**Affiliations:** 1 Faculty of Health Sciences, University of León, 24071 León, Spain; 2 Department of Health Sciences, European University Miguel de Cervantes, 47012 Valladolid, Spain; 3 Research Center on Physical Disability, ASPAYM Castilla y León, 47008 Valladolid, Spain; 4 Multiple Sclerosis Association of Palencia, 34004 Palencia, Spain; 5 Physiotherapy Department, Hospital of Cabueñes, University of Oviedo, 33394 Gijón, Spain; 6 Faculty of Physiotherapy and Nursing, University of Leon, 24071 León, Spain; 7 Physiology Department, University of the Basque Country, 48940 Leioa, Spain

**Keywords:** physical training, activities of daily living, Modified Fatigue Impact Scale

## Abstract

Background: Multiple sclerosis (MS) is a chronic autoimmune disease of the central nervous system that leads to a great deterioration in the quality of life. Objective: We aimed to assess the effectiveness of two individual programs, one based on transcranial direct current stimulation (tDCS) and another based on the effect of physical exercise on fatigue and quality of life in patients with MS. Methods: A total of 12 patients with relapsing–remitting and progressive secondary MS participated. Fatigue and quality of life were assessed before and after intervention. The exercise program and tDCS were carried out over a 4-week period, with a washout period of 5 months. Results: The results show significant improvements in the different quality of life subscales after the application of tDCS, activities of daily living (r = 0.625; *p* = 0.037) (g = 0.465), psychological well-being (r = 0.856; *p* = 0.004) (g = 0.727) and coping (r = 0.904; *p* = 0.18) (g = 0.376), and in those after the application of exercise, activities of daily living (r = 0.853; *p* = 0.003) (g = 0.570) and psychological well-being (r = 0.693; *p* = 0.041) (g = 0.417). After the application of both therapies, more than 50% of the subjects did not have a positive fatigue score on the MFIS scale. Conclusion: The major findings suggest that the application of both therapies produces a beneficial effect with significant improvements in the quality of life of this sample.

## 1. Introduction

Multiple sclerosis (MS) is a chronic autoimmune disease of the central nervous system, with effects including demyelination, axonal loss and inflammatory episodes, making MS the most common cause of neurologic disability in young adults in the Western world [1,2]. Its distribution and prevalence are heterogeneous, and the global median prevalence is 33 per 100,000 people. Generally, young adults are the most affected, and the disease is more frequent in the female sex (3:1) [3,4].

Motor and cognitive dysfunctions are frequent in this type of patients. The limitation of activities, the onset of psychiatric disorders and fatigue cause severe disability, which worsens the physical condition, mobility and quality of life. Quality of life is more limited than in the general population or in those with other chronic diseases, and the decline is progressive in at least one-third of patients after diagnosis [5,6,7].

The worsening of symptoms in this population is associated with lower levels of physical activity, independently of other factors such as depression, neurological disability or the course of the disease, which have a serious impact on quality of life [8]. Therefore, the application of a concurrent physical exercise program, such as the one applied by Grazioli [9], improves the quality of life as well as other factors that affect it, such as the severity of the illness, depression and fatigue.

Another beneficial therapy for MS symptoms is the use of transcranial direct current stimulation (tDCS). The application of anodic-type tDCS produces an increase in cortical excitability, changes in the neurotransmission system of glial cells and changes in the state of the cerebral microvasculature and inflammatory processes [10]. In fact, the application of anodic-type tDCS on the left dorsolateral prefrontal cortex (DLPFC) shows a significant decrease in fatigue, but only the Mortezanejad [11] study has assessed its impact on quality of life, reporting improvements that were maintained up to 4 weeks after intervention.

The current evidence on MS shows a tendency toward improvement in quality of life after the application of an exercise program or anodic tDCS on the DLPFC area. However, such evidence is insufficient to prove these treatments’ effectiveness, and it is important to consider that most studies apply a very small number of sessions of tDCS to an MS population. From this point of view, it is important and necessary to carry out a study with objectives that focuse on the analysis of quality of life after the application of the two interventions. Our hypothesis is that subjects receiving these treatments will show an improvement in quality of life and in the variable of fatigue.

## 2. Materials and Methods

### 2.1. Study Design

A cross-over design was used to carry out this study. On 2 March 2020, the tDCS was applied to the first participant; however, due to health measures imposed by COVID-19, the study was discontinued. Data collection was resumed on 8 June 2020, and this first period ended on 28 August 2020. After a washout period of 5 months, the exercise program was implemented following the guidelines of Muñoz et al. (2022) [12], and data collection was completed on 19 April 2021.

### 2.2. Participants

A sample size calculation was carried out by calculating the difference between dependent groups utilizing the G*Power-3.1.9.2 software (G*Power©, Dusseldorf University, Dusseldorf, Germany). The predetermination of sample size was calculated for α-error of 0.05 and Β error of 0.20. However, after the COVID-19 pandemic, many of the people with MS who attended the rehabilitation centers where the sample was recruited stopped attending due to caution or fear, which made recruitment difficult. For this reason, a crossover design was chosen. In addition, our sample is very small; for this reason, the findings from the patients we present must be considered as preliminary results, and the study should be considered as a pilot study.

In this study, 15 patients participated (6 females and 9 males), but 3 of them were excluded. The reasons for exclusion were as follows: the first one had a surgical intervention, the second presented with COVID-19 and the third was hospitalized due to exacerbation of the disease. The sample was recruited at the Palencia head office of Aspaym Castilla y León and at the Multiple Sclerosis Association of the city of Palencia.

The inclusion criteria followed in this study were a diagnosis of multiple sclerosis (no type of MS was excluded), a score of 38 points or more indicating the presence of fatigue on the Modified Fatigue Impact Scale (MFIS) [13], ability to walk at least 20 m without rest and age of older than 18 years. However, patients were excluded if they had any disease that could affect muscle function, along with those with a cardiovascular risk profile, respiratory disease or other disturbances that could interfere with the exercise program. Those who were pregnant or who did not have a good understanding of and good writing skills in Spanish were also excluded.

### 2.3. Ethical Considerations

The study was approved by the legal ethical committee of the University of León ULE-010-2020. The principles described in the Declaration of Helsinki were followed. An information sheet was given describing the study, including its risks and benefits. This was also explained verbally, in case there were any doubts, including that participants may abandon the intervention at any time. All participants agreed to and signed the written informed consent form prior to enrollment into the study. All participants consented to the publication of identifiable details, which may include photograph(s), video, case history and/or personal details in an online open-access publication.

### 2.4. Interventions

#### 2.4.1. Transcranial Direct Current

The HDCstim stimulator (Newronika, Milán, Italy) was used for the application of tDCS: #HS0042/01-13; HDcel: #HE0021/02-13. Direct current stimulation was distributed with 2mA of current intensity in 35cm2 sponge electrodes. During the session, the current was increased during the first 15 s to a maximum of 2mA that was maintained throughout the 20 min stimulation session.

**Figure 1:**
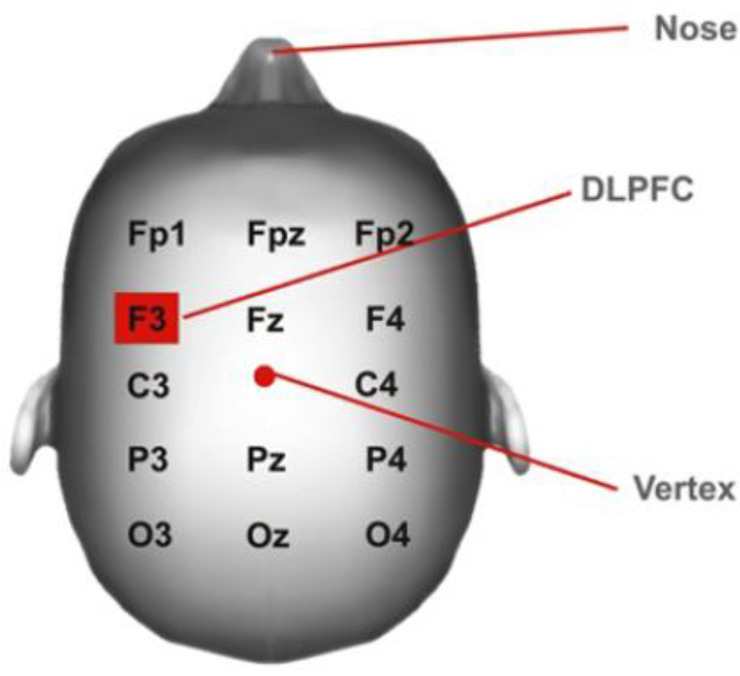
DLPFC (dorsolateral prefrontal cortex) position.

An adjustable head mesh was used to fix the stimulation electrodes in place, and the points of application were determined with the 10–20 EEG system, using the protocol described by DaSilva [14] (Figure 1), as this has been proven to be a useful and cost-effective method to localize the cortical areas. The cathode was laid in the right supraorbital cortex, while the anode was laid in the left DLPFC region (F3 according to the 10–20 EEG system). A total of 10 sessions, 20 min in duration, over the course of 4 weeks were applied by a specialized physiotherapist.

#### 2.4.2. Exercise Program

The exercise program, applied by a specialized physiotherapist, consisted of both strength and aerobic training. Current evidence shows that this type of training is more effective than others for the treatment of fatigue in MS [15,16]. The exercise program was distributed over 4 weeks and conformed to the recommendations that exist for the development of exercise programs in this group of patients [16,17,18,19]. The exercise plan was carried out over 4 weeks, where the sessions progressively increased in intensity (Table A1 and Table A2). The strength and aerobic training sessions were performed on different days according to the recommendations established for this population group.

The strength training was carried out in a circuit. In each circuit, there were 6 exercises of pushing and pulling exercises of the upper and lower limbs, trunk and pelvic girdle. Two circuits were designed that require working the same muscle groups, but in different positions, thus adapting the circuit to facilitate its execution for subjects with some functional limitation and involving the same muscular activity. Moreover, the repetitions and the rest time between exercise and circuits were set in the program. The intensity used for the aerobic training was moderate, which corresponds to a level 3–5 on the rating scale of perceived exertion (RPE). Motomed kinesio therapeutic equipment^®^ (RECK-Technik GmbH & Co. KG, Medizintechnik, Reckstraße 1–5, D-88422 Betzenweiler, Germany) or a static bike were used, depending on the participant’s preference.

### 2.5. Outcome Measures

The primary outcome is the assessment of quality of life after the application of the two treatments. The secondary outcome is to study the relationship between quality of life and sociodemographic, clinical and anthropometric variables, as well as to evaluate fatigue after the application of the two treatments.

Quality of life was assessed before and after each intervention using the Spanish version of the Multiple Sclerosis International Quality of Life (MusiQoL) questionnaire. It consists of 31 items describing 9 dimensions: activities of daily living, psychological well-being, symptomatology, social and family relationships, relationship with the health care system, intimate/sexual life, coping and rejection. The questionnaire uses a 6-point Likert scale, and, for each patient, the score of each dimension is obtained by taking the mean of the scores of the items of each dimension. All dimension scores are linearly translated to a scale from 0 to 100, and negatively marked scores are inverted so that higher scores indicate a higher level of quality of life. The global index score is computed as the mean of the dimension scores. The scale shows good construct validity, internal consistency, reproducibility and reliability [20,21].

Physical activity was evaluated at baseline using the Spanish version of the International Physical Activity Questionnaire Short Form (IPAQ-SF). The reliability and validity have been investigated and analyzed in different countries and contexts and with different types of populations, including in MS patients. Frequency and duration of vigorous, moderate and walking physical activity is measured by this questionnaire during a 7-day period. The respective frequencies and durations were initially multiplied, and the resulting volumes were later multiplied to obtain the METs, by 8 in the case of vigorous activity, 4 in the case of moderate activity and 3.3 in the case of walking [22,23].

The Kurtzke Expanded Disability Status Scale (EDSS) was used to assess neurological involvement and disability. This variable was investigated in the first data collection. It consists of neurological examination findings and includes 20 grades on a scale from 0 (normal examination) to 10 (death due to MS), with 0.5-point intervals. Patients are assessed with regard to the neurological examination and clinical history for each functional system, and, then, taking into account the ability to walk [24,25], an overall score is obtained.

The Spanish version of the Modified Fatigue Impact Scale (MFIS) was used to assess fatigue before and after each intervention. This questionnaire uses a multidimensional approach and consists of 21 items distributed in 3 subscales. According to the frequency with which the symptom has occurred, the patient will respond to each item regarding the last week. The final scores ranged from 0 to 84, and a score of 38 was established as the cutoff point to indicate the presence of fatigue [13,26].

### 2.6. Procedure

First of all, the study information sheet was distributed, answering any queries presented by the participants. Then, all participants signed the informed consent form.

Afterwards, each participant was given a registration form with basic information to check compliance with the inclusion and exclusion criteria of the study and, thus, determine their ability to complete the self-administered questionnaires. This part had an estimated duration of 15 min.

Consecutively, tDCS was applied over 10 sessions of 20 min in duration over the course of 4 weeks. All participants were stimulated from the beginning. Fatigue and quality of life were measured again with the same questionnaires after the application of the intervention.

A stabilization period of 5 months was used to avoid affecting results obtained after the first intervention. In our case, we selected 5 months because the literature determines that the benefits of the treatment last up to 3 weeks after the application of tDCS, when it is applied for 5 days. In a study by Ferrucci [27], an intensity of 1.5mA was applied for 15 min in the primary motor region (M1) for 5 days. Since it has been shown that the effect of tDCS is cumulative and that this effect is necessary to generate the appropriate adaptations for the desired objective, a long washout period was chosen to prevent these effects from interfering with those of the other therapy.

After this period, fatigue and quality of life data were gathered again, and a concurrent training program was performed for a period of 4 weeks. After this time, fatigue and quality of life data were gathered for the last time. Compliance with attendance at the interventions was assessed by means of an attendance control chart.

### 2.7. Statistical Analysis

For statistical analysis, the Statistical Package for Social Sciences (SPSS 21, SPSS Inc., Chicago, IL USA) software package was used. Descriptive statistics were used in the data analysis to show the data for continuous variables presented as ± standard deviation (SD) and relative frequency (percentage) for categorical variables. The Shapiro–Wilk test was used to evaluate the normality, and the result indicated that not all of them met normality, so non-parametric tests were used for the statistical calculation. In addition, a Wilcoxon signed-rank test was used to analyze the results in fatigue and quality of life after the application of exercise and tDCS. 

The results for the indicators of fatigue and quality of life were analyzed by Friedman post hoc Dunn test.

The effect size was calculated to express the magnitude of the differences between the samples. For this purpose, Hedges’ g (scale: 0–1) was used, which sets the effect size as small (0.2), medium (0.5) or large (0.8) [28].

The significance level for all tests was set at *p* < 0.05.

## 3. Results

**Figure 2:**
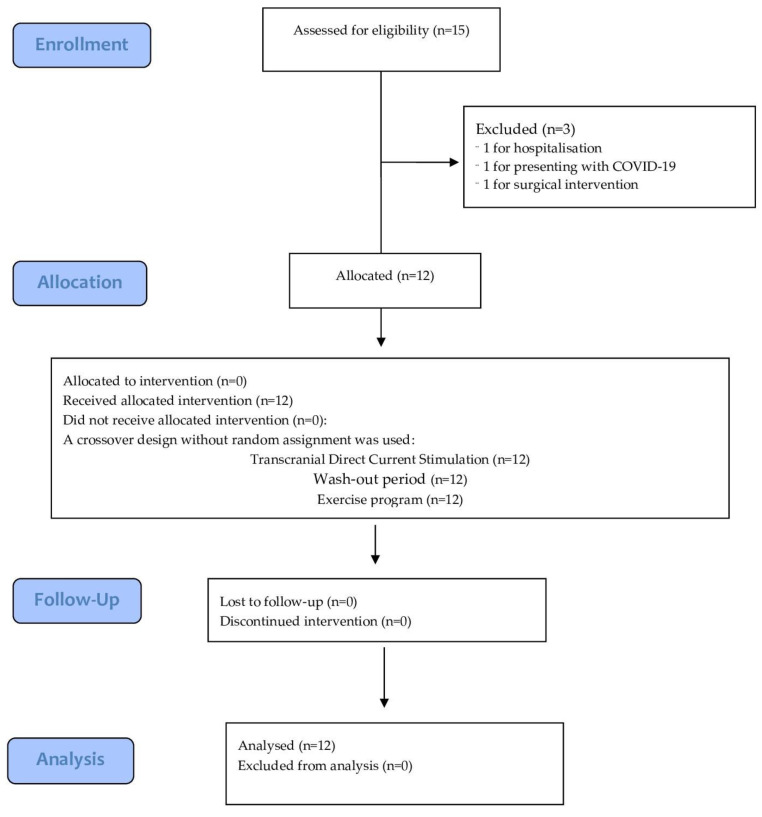
CONSORT flowchart diagram.

The initial characteristics of the participants are shown in Table 1, and the flowchart is shown in Figure 2.

Our study shows that, after the implementation of both therapies, there is a significant improvement in the fatigue and quality of life scales. This suggests that tDCS and exercise may be effective in improving quality of life and fatigue in this sample (Table 2).

Furthermore, the effect size for these outcomes is moderate, which indicates the clinical relevance of the interventions.

Likewise, for the quality-of-life scale, we found significant improvements and clinical changes after the application of tDCS (*p* = 0.015) (g = 0.646) and after exercise (*p* = 0.003) (g = 0.56). Moreover, statistical analysis shows that there have been significant changes after the application of the tDCS in the subscales of activities ADL (*p* = 0.037) (g = 0.465), PWB (*p* = 0.004) (g = 0.727) and COP (*p* = 0.18) (g = 0.376). After the exercise program, significant changes were found in the subscales of ADL (*p* = 0.003) (g = 0.570) and PWB (*p* = 0.041) (g = 0.417).

**Figure 3:**
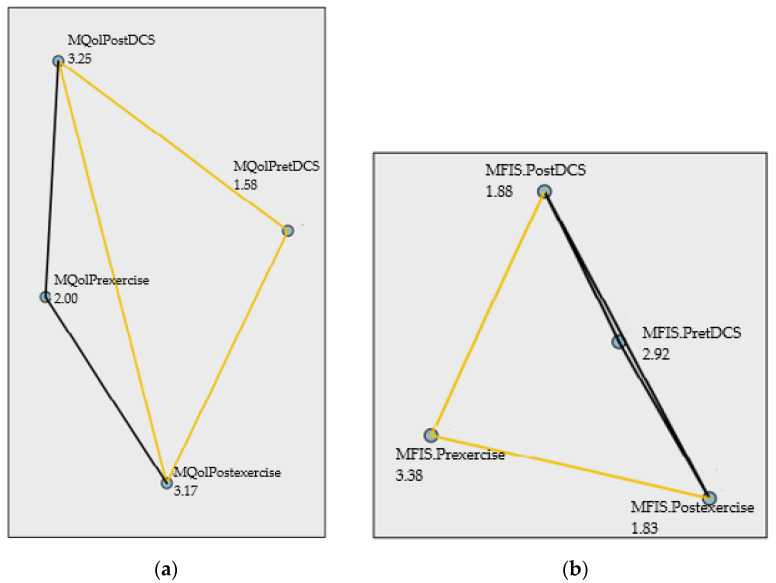
Pairwise comparisons Friedman’s test. (**a**) Multiple Sclerosis International Quality of Life (MusiQoL). (**b**) Modified Fatigue Impact Scale (MFIS).

Furthermore, the Friedman results showed that all variables improved in the tDCS group (Friedman´s test X^2^= 8.33, *p*= 0.002, N = 12), with a significant difference between after tDCS treatment and before treatment tDCS (*p*= 0.009). They also improved in the exercise group (Friedman´s test X^2^= 13.44, *p*= 0.004, N = 12) with a significant difference between after exercise treatment and before treatment exercise (*p*= 0.021), as shown in Figure 3.

Finally, we observed that there were significant changes in fatigue when applying the tDCS (*p* = 0.028) (g = 0.525) and when applying the exercise program (*p* = 0.003) (g = 0.742). Moreover, more than 50% of the subjects did not have a positive fatigue score on the MFIS scale after the application of the therapies.

## 4. Discussion

The results of this study suggest that tDCS and an exercise program may be effective in improving quality of life and fatigue in subjects with MS. Moreover, both variables improved in the groups, with significant differences over time. However, it is important to note that given the sample size and the type of design, our results have to be interpreted with caution.

Quality of life is a multidimensional construct and has special importance in the assessment of MS because it is considered more impaired than in other chronic diseases [29]. In addition, because of the impact of fatigue on quality of life, the studies that focus their therapy on improving quality of life orient the therapies toward the treatment of fatigue symptomatology. For this reason, we thought it was important to analyze the improvement in fatigue after the application of the treatments, so that our results indicate that both the application of the exercise and the tDCS produce beneficial effects.

The current pharmacological treatments are unsatisfactory in many patients because they have modest benefits and numerous side effects. This is perhaps why the combination of pharmacological and other types of therapies may provide the optimal management of the disease and the symptomatology of fatigue [30,31,32,33,34]. Accordingly, the use of physical exercise has the capacity to reverse the consequences of inactivity in these patients and can also modify the anti-inflammatory effect of the disease. This demystifies the belief that the practice of physical activity and the corresponding rise in body temperature could be detrimental for this disease, resulting in a relapse in symptoms [18,35,36].

Despite the benefits of exercise, there is sedentary behavior present in our sample because the mean sitting time spent by the subjects analyzed is 7.77 h, while the mean walking time on a normal day is reduced to 51 min; according to the IPAQ questionnaire, only four of the participants maintain high levels of activity. Therefore, it is important to take into account that sedentary behavior is associated with high levels of mortality and morbidity in the general population and with disability and fatigue in MS [37,38,39].

In our study, the practice of concurrent training shows significant improvements in fatigue and a higher score on the quality of life scale and its various subscales. In particular, in the subscales of activities of daily living and psychological well-being, significant changes were noted. Along the same lines, we found improvements in the same subscales of the MusiQoL questionnaire as in Tarkci’s study [40], comparing a 12-week training program including stretching, balance, core exercises and functional activities with a control group. Performing calisthenics also reportedly brings a significant improvement on the MusiQoL questionnaire [41]. Both studies performed a 12-week exercise program, although we obtained similar results with a 4-week program. It has been shown that practicing a concurrent training program is more effective than strength training and task-oriented training. If we focus on resistance training, several authors support that it is not a consistent measure of fatigue reduction on its own, and, therefore, it is not a consistent measure of quality of life improvement. In addition, one of the reasons why our 4-week exercise program has shown similar results may be due to a better type of training being chosen. Moreover, the intensity of our program is better delimited, since it has been shown that a moderate intensity is ideal in this population. To delimit the intensity of the exercise program, we have different options: the application of 50–70% of the maximum volume of oxygen (VO2 max), 80–60% of the maximum cardiac capacity or a submaximal effort that would correspond to a score of 11–14 of the Borg scale [15,16,19]. However, we must bear in mind that these studies, like ours, include subjects with different types of MS and of different ages. Even so, the beneficial effect of physical exercise on this population has been demonstrated, although in the future it could be interesting to study whether there is a specific type of training that benefits each type of MS.

According to our data, a significant improvement in quality of life and a clinically relevant improvement in the fatigue variable are observed after the application of tDCS. There are several areas of application of this therapy that have been shown to be effective for improvement in fatigue in MS. For example, Cancelli’s group [42] applied the stimulation on the primary somatosensory area, reducing fatigue by 42% after stimulation. Meanwhile, Chalah’s group [43] applied the stimulation on the right posterior parietal lobe, with beneficial effects on fatigue, anxiety and depression. It is true that the area of preference for stimulation is the left DLPFC area, which is the one chosen in this study. One of the reasons for this is that it has been shown to be dose-dependent, which is why we have applied a total of 10 sessions. On the other hand, this stimulation area has been shown to have significant effects both in the decrease in fatigue and in the increase in the quality of life, maintained even 4 weeks after the intervention [11,44,45].

Few articles assessed the quality-of-life scale after the application of tDCS, and none of them used the MusiQoL questionnaire. Only Marzieh’s [11] study evaluated it after application in the left DLPFC area; the other authors used stimulation in the primary motor area, as their objectives were focused on pain. Marzieh’s group used the Multiple Sclerosis Specific Quality of Life Questionnaire (MSQOL-54), obtaining significant improvements that are maintained after stimulation.

Currently, there is no research where tDCS is applied over the left DLPFC area, compared or combined with an exercise program to assess quality of life in MS or other neurological pathologies. Our results indicate that both therapies are effective in improving quality of life, so the application of tDCS in the DLPFC area may have interesting results in MS, without forgetting the cumulative effect of tDCS in order to induce reliable and persistent changes. However, we cannot suggest that one therapy is superior to another.

### 4.1. Practical Application

Quality of life is a complex assessment due to its multifactorial nature, where clinical, psychological and social data converge and where the patient’s perception takes on special relevance. It is, therefore, of particular importance to assess whether the treatments applied in this population benefit this aspect. In this case, both the application of the exercise program and the application of tDCS have shown beneficial effects in quality of life and in several of its subscales. Therefore, we could suggest that the application of concurrent training, which not only focuses its work on the knee musculature but also on the secondary and stabilizing muscles of the trunk, hip and upper limb, could be beneficial in these subjects, regardless of their age and type of sclerosis, as all of them significantly increased their score on the quality of life scale. On the other hand, the benefits suggested by the application of tDCS on the left DLPFC area on quality of life may be interesting to study in the future, along with its effectiveness and potential in larger samples, and to check, if appropriate, if the application on this area shows better results than in other areas, such as the primary motor cortex. Nevertheless, in line with the suggestions of other studies, such as the review by Giuseppina et al. [46], the combination of tDCS and exercise with prolonged treatment with multiple sessions would be optimal to generate measurable benefits.

### 4.2. Limitations

One of the limitations of our study is the small sample size; for this reason, the findings from the patients we present must be considered as preliminary results, and the study should be considered as a pilot study. In addition, our data should be treated with caution as the sample is limited, and we did not have a long follow-up period. Future research with larger samples is necessary; similarly, randomized clinical trials are needed to accurately determine the efficacy and effectiveness of the treatment.

## 5. Conclusions

Major findings suggest that both the implementation of an exercise program and the application of tDCS produce a beneficial effect with significant improvements in quality of life in this sample. In the same way, it could be suggested that adding tDCS could provide further improvements to physical therapy, but further research with a larger sample and sham stimulation as a control is needed.

## Figures and Tables

**Table 1:** Participant’s baseline demographic and clinical characteristics.

VARIABLES	N [MIN–MAX]; MEAN ± (SD)	FREQUENCY (%)
Age	12 [35–66]; 48.08 ± 8.55	
Years of diagnosis	12 [0.8–28]; 16.65 ± 7.44	
Outbreaks per year	11 [0–2]; 0.36 ± 0.67	
Walking time (minutes)	9 [0.0–120]; 51.11 ± 41.06	
Sitting time (minutes)	9 [0–960]; 466.667 ± 304.13	
Type of sclerosis		
Relapsing–remitting Progressive secondary		7 (58.3%)
		5 (41.7%)
Outbreak intensity		
Mild Moderate Intense No outbreaks		2 (18.2%)
		1 (9.1%)
		1 (9.1%)
		7 (63.6%)
Medical recommendation		
Physical activity		6 (11.3%)
Other		1 (8.3%)
No recommendation		5 (41.7%)
Fatigue medication		
Yes No		5 (41.7%)
		7 (58.3%)
Type of medication fatigue		
LioresalLioresal + Avonex Lioresal + Rebif 44 Other medication No medication		1 (9.1%)
		1 (9.1%)
		1 (9.1%)
		2 (16.7%)
		7 (58.3%)
Rehabilitation		
Yes		11 (91.7%)
No		1 (8.3%)
Intensity rehabilitation		
Occasional		5 (41.7%)
Periodic		7 (58.3%)
Exercise habits		
Occasional Regularly		2 (16.7%)
		10 (83.3%)
Education level		
Primary education Secondary studies Vocational training University studies		1 (8.3%)
		1 (8.3%)
		6 (50%)
		4 (33.3%)
Employment situation		
Homemaker Part-time employee Full-time employee Retired Permanently disabled		1 (8.3%)
		1 (8.3%)
		5 (41.7%)
		3 (25%)
		2 (16.7%)

**Table 2:** Pre–post tDCS and exercise through Wilcoxon test: quality of life and fatigue.

	Pre tDCS Median [Range]	Post tDCS Median [Range]	*p*	Size Effect Hedges’ g	Pre-Exercise Median [Range]	Post-Exercise Median [Range]	*p*	Size Effect Hedges’ g
MQOL	68.04 [25.28]	75.19 [32.23]	0.015 *	0.646	70.07 [28.99]	74.94 [27.2]	0.003 **	0.56
MQOLADL	58.9 [6.25]	64.80 [6.55]	0.037 *	0.465	57.73 [1.5]	68.09 [6.95]	0.003 **	0.570
MQOLPWB	66.24 [3]	80.42 [6.82]	0.004 **	0.727	65 [2]	72.5 [4.33]	0.41 *	0.417
MQOLSYM	72.5 [4]	77.5 [5.8]	0.438	0.258	72.5 [6.17]	74.58 [4.42]	0.625	0.122
MQOLSOREL	76.93 [4.88]	78.31 [5.92]	0.413	0.055	84.98 [2]	87.2 [3.67]	0.336	0.120
MQOLRFAREL	81.65 [6.25]	83.32 [3.92]	0.44	0.066	88.88 [2.3]	90.53 [3.4]	0.102	0.104
MQOLSEXLIFE	73.75 [5]	75 [3.25]	0.865	0.045	73.33 [2]	77.5 [2.67]	0.257	0.129
MQOLCOP	50.83 [3]	61.67 [5.25]	0.018 *	0.376	51.67 [5.75]	56.66 [4.79]	0.177	0.266
MQOLREJEC	68.33 [3]	79.17 [6]	0.103	0.475	75 [3.5]	80 [4.08]	0.058	0.221
MQOLREHEALTH	79.41 [3.38]	78.88 [4.83]	0.933	0.036	78.87 [2.3]	82.07 [2.67]	0.269	0.152
MFIS	39.5 [31]	38.5 [45]	0.028 *	0.525	43 [33]	36 [52]	0.003 **	0.742

Non-parameter statistics. Wilcoxon signed-rank test. MQOL: Multiple Sclerosis International Quality of Life. MQoLADL: MusiQoL subscale activities of daily life. MQoLPWB: MusiQoL subscale psychological well-being. MQoLSYM: MusiQoL subscale symptomatology. MQoLSOREL: MusiQoL subscale social relationship. MQoLFAREL: MusiQoL subscale family relationship. MQoLSEXLIFE: MusiQoL subscale sexual life. MQoLCOP: MusiQoL subscale coping. MQoLREJEC: MusiQoL subscale rejection. MQoLREHEALTH: MusiQoL subscale relationship with the health care. MFIS: Modified Fatigue Impact Scale. tDCS: current direct transcranial stimulation. Pre tDCS: treatment before transcranial direct current stimulation (tDCS). Post tDCS: treatment after transcranial direct current stimulation (tDCS). * *p* < 0.05 and ** *p* < 0.001.

## Data Availability

The datasets used and/or analyzed in the current study are available from the corresponding author on reasonable request.

## Appendix A

**Table A1:** Exercise program schedule.

	Week 1	Week 2	Week 3	Week 4
Endurance	1 Session	2 Sessions	2 Sessions	2-3 Sessions
	10 min	15 min	10 min + 10 min (5 min of rest)	15 min + 15 min (5 min of rest)
	3–5 RPE	3–5 RPE	3–5 RPE	3–5 RPE
	MOTOmed/static bike	MOTOmed/static bike	MOTOmed/static bike	MOTOmed/static bike
Strength	2 Sessions (A, B)	2 Sessions (A, B)	3 Sessions (circuit to choose)	3 Sessions (circuit to choose)
	6 Exercise in each circuit	6 Exercise in each circuit	6 Exercise in each circuit	6 Exercise in each circuit
	15 Rep (rest: 2 min)	15 Rep (rest: 2 min)	10 Rep (rest: 3 min)	10 Rep (rest: 3 min)
	2Circuits (rest: 3 min)	2 Circuits (rest: 3 min)	3 Circuits (rest: 5 min)	3 Circuits (rest: 5 min)

Exercise program. A, B refers to exercises specified in the table. RPE = rate of perceived exertion. Min = minutes. MOTOmed = aerobic kinesiotherapy equipment. Rep = repetitions.

**Table A2:** Exercises are used in circuits A and B of the exercise program.

Exercise	Circuit A	Circuit B
MMSS PULL		
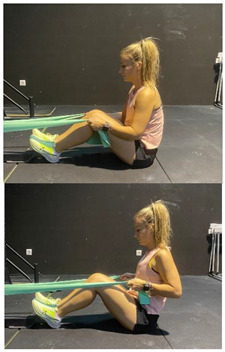
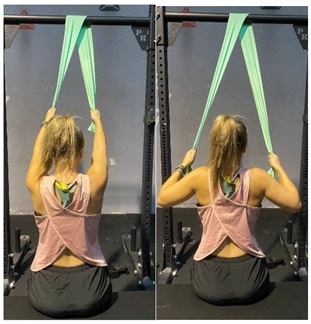
MMSS PUSH		
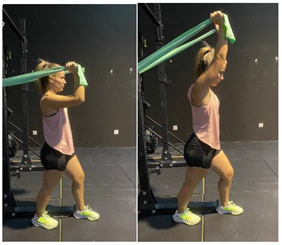
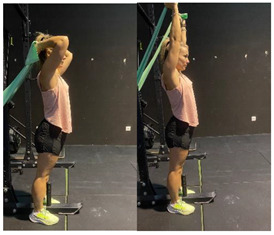
KNEE EXTENSION		
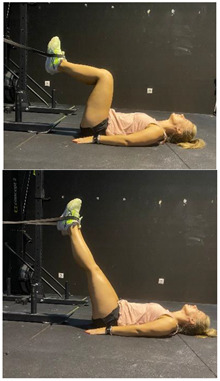
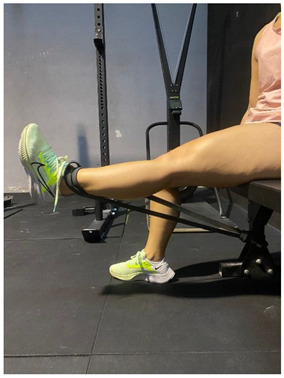
KNEE FLEXION		
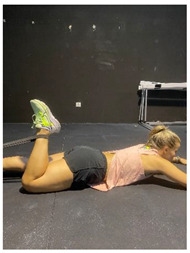
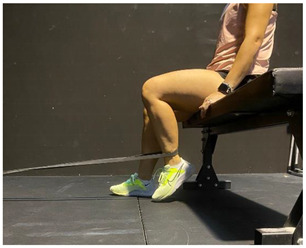
HIP EXTENSION		
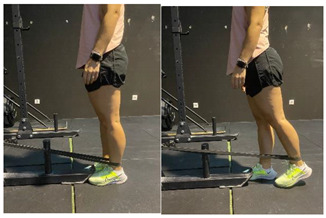
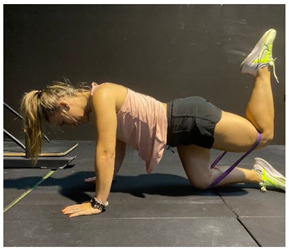
HIP FLEXION		
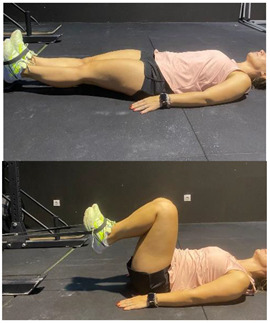
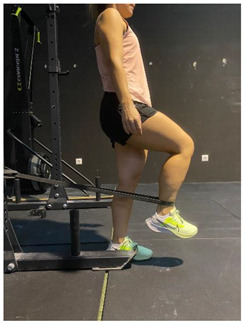
CORE		
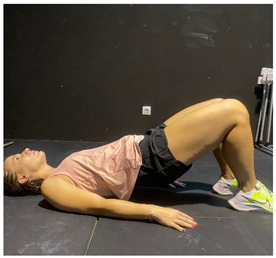
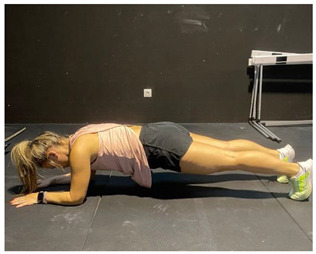

Images of the exercises developed according to the objectives to be achieved. Two different exercises are shown for each objective.
